# Estimation of time-varying reproduction numbers underlying epidemiological processes: A new statistical tool for the COVID-19 pandemic

**DOI:** 10.1371/journal.pone.0236464

**Published:** 2020-07-21

**Authors:** Hyokyoung G. Hong, Yi Li

**Affiliations:** 1 Department of Statistics and Probability, Michigan State University, East Lansing, MI, United States of America; 2 Department of Biostatistics, University of Michigan, Ann Arbor, MI, United States of America; National Institute of Environmental Health Sciences, UNITED STATES

## Abstract

The coronavirus pandemic has rapidly evolved into an unprecedented crisis. The susceptible-infectious-removed (SIR) model and its variants have been used for modeling the pandemic. However, time-independent parameters in the classical models may not capture the dynamic transmission and removal processes, governed by virus containment strategies taken at various phases of the epidemic. Moreover, few models account for possible inaccuracies of the reported cases. We propose a Poisson model with time-dependent transmission and removal rates to account for possible random errors in reporting and estimate a time-dependent disease reproduction number, which may reflect the effectiveness of virus control strategies. We apply our method to study the pandemic in several severely impacted countries, and analyze and forecast the evolving spread of the coronavirus. We have developed an interactive web application to facilitate readers’ use of our method.

## 1 Introduction

Coronaviruses are enveloped single-stranded positive-sense RNA viruses belonging to a broad family of coronaviridae and are widely harbored in animals [1–3]. Most of the coronaviruses only cause mild respiratory infections, but SARS-CoV-2, a newly identified member of the coronavirus family, initiated the contagious and lethal coronavirus disease 2019 (COVID-19) in December 2019 [4, 5]. Since the detection of the first case in Wuhan, the COVID-19 pandemic has evolved into a global crisis within only four months. As of June 30, 2020, the virus has infected more than 10 million individuals, caused about 518,000 deaths [6], and altered the life of billions of people.

The pandemic has been closely monitored by the international society. For example, the World Health Organization (WHO) and Johns Hopkins University’s Coronavirus Resource Center [6] have, since the outbreak, reported the daily numbers of infectious and recovered cases, and deaths for nearly every country. The governmental websites of many counties, such as Australia, the US, Singapore, also have been tracking these numbers starting from various time points. These websites have become valuable resources to help advance the understanding of spread of the virus. We have access to a time-series data repository on GitHub (https://github.com/ulklc/covid19-timeseries), which consolidates and updates information obtained from these data sources. Our data analysis is based on the data obtained from this GitHub data repository.

Much effort has been devoted by the affected countries to battling the disease. However, the crisis has not been over, with new infections detected every day. To forecast when the pandemic gets controlled and evaluate the effects of virus control measures, it is imperative to develop appropriate models to describe and understand the change trend of the pandemic [7–10].

The susceptible-infectious-removed (SIR) model was utilized to explain the rapid rise and fall of the infected individuals from the epidemics of severe acute respiratory syndrome (SARS), influenza A virus subtype (H1N1) and middle east respiratory syndrome (MERS) [11–15]. The key idea is to divide a total population into three compartments: the susceptible, *S*, who are healthy individuals capable of contracting the disease; the infectious, *I*, who have the disease and are infectious; and the removed, *R*, who have recovered from the disease and gained immunity or who have died from the disease [16]. The model assumes a one-way flow from susceptible to infectious to removed, and is reasonable for infectious diseases, which are transmitted from human to human, and where recovery confers lasting resistance [17]. SIR models originated from the Kermack-McKendrick model [18], consisting of three coupled differential equations to describe the dynamics of the numbers in the *S*, *I*, and *R* compartments, which tend to fluctuate over time. For example, the number of infectious individuals increases drastically at the start of the epidemic, with a surge in susceptible individuals becoming infectious. As the epidemic develops, the number of infectious individuals decreases when more infectious individuals die or recover than susceptible individuals become infectious. The epidemic ends when the infectious compartment ceases to exist [16, 18].

SIR models and the modified versions, such as susceptible-exposed-infectious-recovered model (SEIR), were applied to analyze the COVID-19 outbreak [19–23]. Many of these models assume constant transmission and removal rates, which may not hold in reality. For example, as a result of various virus containment strategies, such as self-quarantine and social distancing mandates, the transmission and removal rates may vary over time [24].

Recently, a number of researchers [25–27] considered time-dependent SIR models adapted to the dynamical epidemiological processes evolving over time. However, few considered random errors in reporting, such as under-reporting (e.g. asymptomatic cases or virus mutation) or over-reporting (e.g. false positives of testing), or characterized the uncertainty of predictions.

Poisson models naturally fit count data [28]. Several works [29–31] used Poisson distributions to model *I* and *R* from frequentist or Bayesian perspectives; however, most of the works only considered constant transmission and removal rates. How to extend these works to accommodate time-dependent rates remains elusive.

We propose to adopt a Poisson model to estimate the time-varying transmission and removal rates, and understand the trends of the pandemic across countries. For example, we can predict the number of the infectious persons and the number of removed persons at a certain time for each country, and forecast when the curves of cases become flattened.

An important epidemiological index that characterizes the transmission potential is the basic reproduction number, $R_{0}$, defined as the expected number of secondary cases produced by an infectious case [32–34]. Our model leads to a temporally dynamical $R_{0}$, which measures at a given time how many people one infectious person, during the infectious period, will infect [35]. This may help evaluate the quarantine policies implemented by various authorities. A recent work [35] demonstrated that $R_{0}$ is likely to vary “due to the impact of the performed intervention strategies and behavioral changes in the population”.

The merits of our work are summarized as follows. First, unlike the deterministic ODE-based SIR models, our method does not require transmission and removal rates to be known, but estimates them using the data. Second, we allow these rates to be time-varying. Some time-varying SIR approaches [27] directly integrate into the model the information on when governments enforced, for example, quarantine, social-distancing, compulsory mask-wearing and city lockdowns. Our method differs by computing a time-varying $R_{0}$, which gauges the status of coronavirus containment and assesses the effectiveness of virus control strategies. Third, our Poisson model accounts for possible random errors in reporting, and quantifies the uncertainty of the predicted numbers of susceptible, infectious and removed. Finally, we apply our method to analyze the data collected from the aforementioned GitHub time-series data repository. We have created an interactive web application (https://younghhk.shinyapps.io/tvSIRforCOVID19/) to facilitate users’ application of the proposed method.

## 2 A Poisson model with time-dependent transmission and removal rates

We introduce a Poisson model with time-varying transmission and removal rates, denoted by *β*(*t*) and *γ*(*t*). Consider a population with *N* individuals, and denote by *S*(*t*), *I*(*t*), *R*(*t*) the true but unknown numbers of susceptible, infectious and removed, respectively, at time *t*, and by *s*(*t*) = *S*(*t*)/*N*, *i*(*t*) = *I*(*t*)/*N*, *r*(*t*) = *R*(*t*)/*N* the fractions of these compartments.

### 2.1 Time-varying transmission, removal rates and reproduction number

The following ordinary differential equations (ODE) describe the change rates of *s*(*t*), *i*(*t*) and *r*(*t*):

$$\begin{array}{ccc} \frac{ds(t)}{dt} & = & -\beta (t)s(t)i(t), \end{array}$$ (1)

$$\begin{array}{ccc} \frac{di(t)}{dt} & = & \beta (t)s(t)i(t)-\gamma (t)i(t), \end{array}$$ (2)

$$\begin{array}{ccc} \frac{dr(t)}{dt} & = & \gamma (t)i(t), \end{array}$$ (3)

with an initial condition: *i*(0) = *i*_0_ and *r*(0) = *r*_0_, where *i*_0_ > 0 in order to let the epidemic develop [36]. Here, *β*(*t*) > 0 is the time-varying transmission rate of an infection at time *t*, which is the number of infectious contacts that result in infections per unit time, and *γ*(*t*) > 0 is the time-varying removal rate at *t*, at which infectious subjects are removed from being infectious due to death or recovery [33]. Moreover, *γ*^−1^(*t*) can be interpreted as the infectious duration of an infection caught at time *t*[37].

From (1)–(3), we derive an important quantity, which is the time-dependent reproduction number

$$\begin{array}{c} R_{0}\left(t\right)=\frac{\beta (t)}{\gamma (t)}. \end{array}$$

To see this, dividing (2) by (3) leads to

$$\begin{array}{c} R_{0}\left(t\right)=\frac{1}{s(t)}\{\frac{di}{dr}\left(t\right)+1\}, \end{array}$$ (4)

where (*di*/*dr*)(*t*) is the ratio of the change rate of *i*(*t*) to that of *r*(*t*). Therefore, compared to its time-independent counterpart, $R_{0}\left(t\right)$ is an instantaneous reproduction number and provides a real-time picture of an outbreak. For example, at the onset of the outbreak and in the absence of any containment actions, we may see a rapid ramp-up of cases compared to those removed, leading to a large (*di*/*dr*)(*t*) in (4), and hence a large $R_{0}\left(t\right)$. With the implemented policies for disease mitigation, we will see a drastically decreasing (*di*/*dr*)(*t*) and, therefore, declining of $R_{0}\left(t\right)$ over time. The turning point is *t*_0_ such that $R_{0}\left(t_{0}\right)=1,$ when the outbreak is controlled with (*di*/*dr*)(*t*_0_) < 0.

Under the fixed population size assumption, i.e., *s*(*t*) + *i*(*t*)+ *r*(*t*) = 1, we only need to study *i*(*t*) and *r*(*t*), and re-express (1)–(3) as

$$\begin{array}{ccc} \frac{di(t)}{dt} & = & \beta (t)i(t)\{1-i(t)-r(t)\}-\gamma (t)i(t),\\ \frac{dr(t)}{dt} & = & \gamma (t)i(t), \end{array}$$ (5)

with the same initial condition.

### 2.2 A Poisson model based on discrete time-varying SIR

As the numbers of cases and removed are reported on a daily basis, *t* is measured in days, e.g. *t* = 1, …, *T*. Replacing derivatives in (5) with finite differences, we can consider a discrete version of (5):

$$\begin{array}{ccc} i(t+1)-i(t) & = & \beta (t)i(t)\{1-i(t)-r(t)\}-\gamma (t)i(t),\\ r(t+1)-r(t) & = & \gamma (t)i(t), \end{array}$$ (6)

where *β*(*t*) and *γ*(*t*) are positive functions of *t*. We set *i*(0) = *i*_0_ and *r*(0) = *r*_0_ with *t* = 0 being the starting date.

Model (6) admits a recursive way to compute *i*(*t*) and *r*(*t*):

$$\begin{array}{ccc} i(t+1) & = & \{1+\beta (t)-\gamma (t)\}i(t)-\beta (t)i(t)\{i(t)+r(t)\},\\ r(t+1) & = & r(t)+\gamma (t)i(t) \end{array}$$ (7)

for *t* = 0, …, *T* − 1. The first equation of (7) implies that *β*(*t*) < *γ*(*t*) or $R_{0}\left(t\right)=\beta \left(t\right)\gamma^{-1}\left(t\right)<1$ leads to that *i*(*t* + 1) < *i*(*t*) or the number of infectious cases drops, meaning the spread of virus is controlled; otherwise, the number of infectious cases will keep increasing.

### 2.3 Estimation and inference

To fit the model and estimate the time-dependent parameters, we can use nonparametric techniques, such as splines [38–43], local polynomial regression [44] and reproducible kernel Hilbert space method [45]. In particular, we consider a cubic B-spline approximation [46].

Denote by **B**(*t*) = {*B*_1_(*t*),…,*B_q_*(*t*)}*_T_* the *q* cubic B-spline basis functions over [0, *T*] associated with the knots 0 = *w*_0_ < *w*_1_ < … < *w*_*q*−2_ < *w*_*q*−1_ = *T*. For added flexibility, we allow the number of knots to differ between *β*(*t*) and *γ*(*t*) and specify

$$\begin{array}{ccc} \log\beta (t) & = & \sum_{j=1}^{q_{1}}\beta_{j}B_{j}\left(t\right),\\ \log\gamma (t) & = & \sum_{j=1}^{q_{2}}\gamma_{j}B_{j}\left(t\right). \end{array}$$ (8)

When $\beta_{1}=\cdots =\beta_{q_{1}}$ and $\gamma_{1}=\cdots =\gamma_{q_{2}}$, the model reduces to a constant SIR model [46]. We use cross-validation to choose *q*_1_ and *q*_2_ in our numerical experiments.

Denote by $\beta =(\beta_{1},\ldots ,\beta_{q_{1}})$ and $\gamma =(\gamma_{1},\ldots ,\gamma_{q_{2}})$ the unknown parameters, by *Z*_*I*_(*t*) and *Z*_*R*_(*t*) the reported numbers of infectious and removed, respectively, and by *z*_*I*_(*t*) = *Z*_*I*_(*t*)/*N* and *z*_*R*_(*t*) = *Z*_*R*_(*t*)/*N*, the reported proportions. Also, denote by *I*(*t*) and *R*(*t*) the true numbers of infectious and removed, respectively at time *t*. We propose a Poisson model to link *Z*_*I*_(*t*) and *Z*_*R*_(*t*) to *I*(*t*) and *R*(*t*) as follows:

$$\begin{array}{ccc} Z_{R}\left(t\right) & ∼ & \text{Pois}\{R(t)\},\\ Z_{I}\left(t\right) & ∼ & \text{Pois}\{I(t)\}. \end{array}$$ (9)

We also assume that, given *I*(*t*) and *R*(*t*), the observed daily number {*Z*_*I*_(*t*), *Z*_*R*_(*t*)} are independent across *t* = 1, …, *T*, meaning the random reporting errors are “white” noise. We note that (9) is directly based on “true” numbers of infectious cases and removed cases derived from the discrete SIR model (6). This differs from the Markov process approach, which is based on the past observations.

With (6), (7) and (8), *R*(*t*) and *I*(*t*) are the functions of ***β*** and ***γ***, since *R*(*t*) = *N* × *r*(*t*) and *I*(*t*) = *N* × *i*(*t*). Given the data (*Z*_*I*_(*t*), *Z*_*R*_(*t*)), *t* = 1, …, *T*, we obtain $\left(\hat{\beta },\hat{\gamma }\right)$, the estimates of (***β*, *γ***), by maximizing the following likelihood

$$\begin{array}{c} L\left(\beta ,\gamma \right)=\prod_{t=1}^{T}\frac{e^{-R(t)}R{\left(t\right)}^{Z_{R(t)}}}{Z_{R}\left(t\right)!}\times \prod_{t=1}^{T}\frac{e^{-I(t)}I{\left(t\right)}^{Z_{I}\left(t\right)}}{Z_{I}\left(t\right)!}, \end{array}$$

or, equivalently, maximizing the log likelihood function

$$\begin{array}{c} \ell \left(\beta ,\gamma \right)=N\sum_{t=1}^{T}\{-r\left(t\right)+z_{R}\left(t\right)\log r\left(t\right)-i\left(t\right)+z_{I}\left(t\right)\log i\left(t\right)\}+C, \end{array}$$ (10)

where *C* is a constant free of ***β*** and ***γ***. See the S1 Appendix for additional details of optimization.

We then estimate the variance-covariance matrix of $\left(\hat{\beta },\hat{\gamma }\right)$ by inverting the second derivative of −ℓ(***β*, *γ***) evaluated at $\left(\hat{\beta },\hat{\gamma }\right)$. Finally, for *t* = 1, …, *T*, we estimate *I*(*t*) and *R*(*t*) by $\hat{I}\left(t\right)=N\hat{i}\left(t\right)$ and $\hat{R}\left(t\right)=N\hat{r}\left(t\right)$, where $\hat{i}\left(t\right)$ and $\hat{r}\left(t\right)$ are obtained from (7) with all unknown quantities replaced by their estimates; estimate *β*(*t*) and *γ*(*t*) by $\hat{\beta }\left(t\right)$ and $\hat{\gamma }\left(t\right)$, obtained by using (8) with (***β*, *γ***) replaced by $\left(\hat{\beta },\hat{\gamma }\right)$; and estimate $R_{0}\left(t\right)$ by ${\hat{R}}_{0}\left(t\right)=\hat{\beta }\left(t\right)/\hat{\gamma }\left(t\right)$.

**Summary of estimation and inference for *β*(*t*), *γ*(*t*)**, $R_{0}\left(t\right)$, *I*(*t*), *R*(*t*)

**Estimation:** Let *N* be the size of population of a given country. The date when the first case was reported is set to be the starting date with *t* = 1, *i*_0_ = *Z*_*I*_(1)/*N* and *r*_0_ = *Z*_*R*_(1)/*N*. The observed data are {*Z*_*I*_(*t*), *Z*_*R*_(*t*), *t* = 1, …, *T*}, obtained from the GitHub data repository website mentioned in the introduction. We maximize (10) to obtain $\hat{\beta }=\left({\hat{\beta }}_{0},{\hat{\beta }}_{1},\ldots ,{\hat{\beta }}_{q_{1}}\right)$ and $\hat{\gamma }=\left({\hat{\gamma }}_{0},{\hat{\gamma }}_{1},\ldots ,{\hat{\gamma }}_{q_{2}}\right)$. The optimal *q*_1_ and *q*_2_ are obtained via cross-validation. We denote by $\hat{\beta }=\left({\hat{\beta }}_{0},{\hat{\beta }}_{1},\ldots ,{\hat{\beta }}_{q_{1}}\right)$ and $\hat{\gamma }=\left({\hat{\gamma }}_{0},{\hat{\gamma }}_{1},\ldots ,{\hat{\gamma }}_{q_{2}}\right)$, based on which we calculate $\hat{\beta }\left(t\right),\hat{\gamma }\left(t\right),{\hat{R}}_{0}\left(t\right),\hat{R}\left(t\right),\hat{I}\left(t\right)$.
**Inference:** The estimated variance-covariance matrix of $\left(\hat{\beta },\hat{\gamma }\right)$, denoted by $\hat{V}\left(\hat{\beta },\hat{\gamma }\right)$, can be obtained by inverting the second derivative of −ℓ(***β*, *γ***) evaluated at $\left(\hat{\beta },\hat{\gamma }\right)$. For each *t*, as $\hat{\beta }\left(t\right)$, $\hat{\gamma }\left(t\right)$, ${\hat{R}}_{0}\left(t\right)$, $\hat{R}\left(t\right)$ and $\hat{I}\left(t\right)$ are smooth functions of $\hat{\beta }$ and $\hat{\gamma }$, we apply the delta method [47] to estimate their variances and obtain the confidence intervals. As an illustration, we compute $\hat{\text{var}}\left(\hat{R}\left(t\right)\right)=\dot{\hat{R}}{\left(t\right)}^{T}\hat{V}\left(\hat{\beta },\hat{\gamma }\right)\dot{\hat{R}}\left(t\right)$ and $\hat{\text{var}}\left(\hat{I}\left(t\right)\right)=\dot{\hat{I}}{\left(t\right)}^{T}\hat{V}\left(\hat{\beta },\hat{\gamma }\right)\dot{\hat{I}}\left(t\right),$ where $\dot{\hat{R}}\left(t\right)$ and $\dot{\hat{I}}\left(t\right)$ are the partial derivative vectors of $\hat{R}\left(t\right)$ and $\hat{I}\left(t\right)$ with respect to $\left(\hat{\beta },\hat{\gamma }\right)$.

## 3 Analysis of the COVID-19 pandemic among severely affected countries

Since the first case of COVID-19 was detected in China, it quickly spread to nearly every part of the world [6]. COVID-19, conjectured to be more contagious than the previous SARS and H1N1 [48], has put great strain on healthcare systems worldwide, especially among the severely affected countries [49]. We apply our method to assess the epidemiological processes of COVID-19 in some severely impacted countries.

### 3.1 Data descriptions and robustness of the method towards specifications of the initial conditions

The country-specific time-series data of confirmed, recovered, and death cases were obtained from a GitHub data repository website (https://github.com/ulklc/covid19-timeseries). This site collects information from various sources listed below on a daily basis at GMT 0:00, converts the data to the CSV format, and conducts data normalization and harmonization if inconsistencies are found. The data sources include

- World Health Organization (WHO): https://www.who.int/
- DXY.cn. Pneumonia 2020: http://3g.dxy.cn/newh5/view/pneumonia.
- BNO News: https://bnonews.com/index.php/2020/02/the-latest-coronavirus-cases/
- National Health Commission of China (NHC): http://www.nhc.gov.cn/xcs/yqtb/list_gzbd.shtml
- China CDC (CCDC): http://weekly.chinacdc.cn/news/TrackingtheEpidemic.htm
- Hong Kong Department of Health: https://www.chp.gov.hk/en/features/102465.html
- Macau Government: https://www.ssm.gov.mo/portal/
- Taiwan CDC: https://sites.google.com/cdc.gov.tw/2019ncov/taiwan?authuser=0
- US CDC: https://www.cdc.gov/coronavirus/2019-ncov/index.html
- Government of Canada: https://www.canada.ca/en/public-health/services/diseases/coronavirus.html
- Australia Government Department of Health: https://www.health.gov.au/news/coronavirus-update-at-a-glance
- European Centre for Disease Prevention and Control (ECDC): https://www.ecdc.europa.eu/en/geographical-distribution-2019-ncov-cases
- Ministry of Health Singapore (MOH): https://www.moh.gov.sg/covid-19
- Italy Ministry of Health: http://www.salute.gov.it/nuovocoronavirus
- Johns Hopkins CSSE: https://github.com/CSSEGISandData/COVID-19
- WorldoMeter: https://www.worldometers.info/coronavirus/

**Fig 1:**
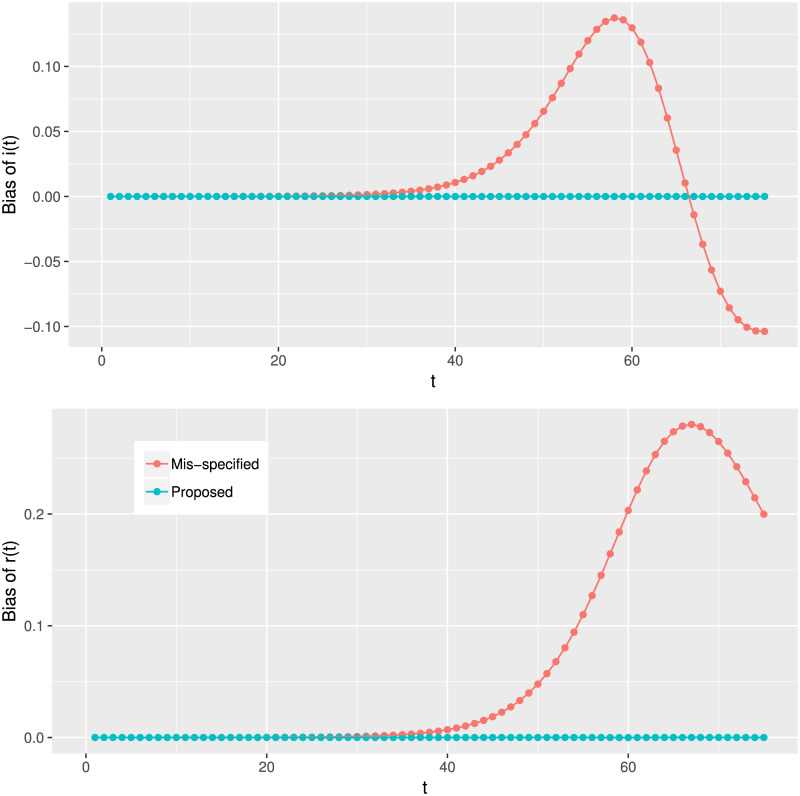
The impact of mis-specification of *i*_0_ and *r*_0_. Plots of the relative biases of $\hat{i}\left(t\right)$ (upper) and $\hat{r}\left(t\right)$ (lower) when $\hat{i}\left(t\right)$ and $\hat{r}\left(t\right)$ are derived 1) by using the ODE framework with the mis-specified initials (“Mis-specified”) and 2) by using proposed model with mis-specified initials (“Proposed”). In the model, the true (*β*, *γ*) = (*e*^−1^, *e*^−1.95^) and (*i*_0_, *r*_0_) = (10^−6^, 10^−6^). These values are roughly equal to the constant estimates of the real situation. The mis-specified initials are set as (5 × 10^−6^, 5 × 10^−6^).

In particular, the current population size of each country, *N*, came from the website of WorldoMeter. Our analyses covered the periods between the date of the first reported coronavirus case in each nation and June 30, 2020. In the beginning of the outbreak, assessment of *i*_0_ and *r*_0_ was problematic as infectious but asymptomatic cases tended to be undetected due to lack of awareness and testing. To investigate how our method depends on the correct specification of the initial values *r*_0_ and *i*_0_, we conducted Monte Carlo simulations. As a comparison, we also studied the performance of the deterministic SIR model in the same settings. Fig 1 shows that, when the initial value *i*_0_ was mis-specified to be 5 times of the truth, the curves of *i*(*t*) and *r*(*t*) obtained by the deterministic SIR model (6) were considerably biased. On the other hand, our proposed model (9), by accounting for the randomness of the observed data, was robust toward the mis-specification of *i*_0_ and *r*_0_: the estimates of *r*(*t*) and *i*(*t*) had negligible biases even with mis-specified initial values. In an omitted analysis, we mis-specified *i*_0_ and *r*_0_ to be only twice of the truth, and obtain the similar results.

Our numerical experiments also suggested that using the time series, starting from the date when both cases and removed were reported, may generate more reasonable estimates.

### 3.2 Estimation of country-specific transmission, removal rates and reproduction numbers

Using the cubic B-splines (8), we estimated the time-dependent transmission rate *β*(*t*) and removal rate *γ*(*t*), based on which we further estimated $R_{0}\left(t\right)$, *I*(*t*) and *R*(*t*). To choose the optimal number of knots for each country when implementing the spline approach, we used 5-fold cross-validation by minimizing the combined mean squared error for the estimated infectious and removed cases.

**Fig 2:**
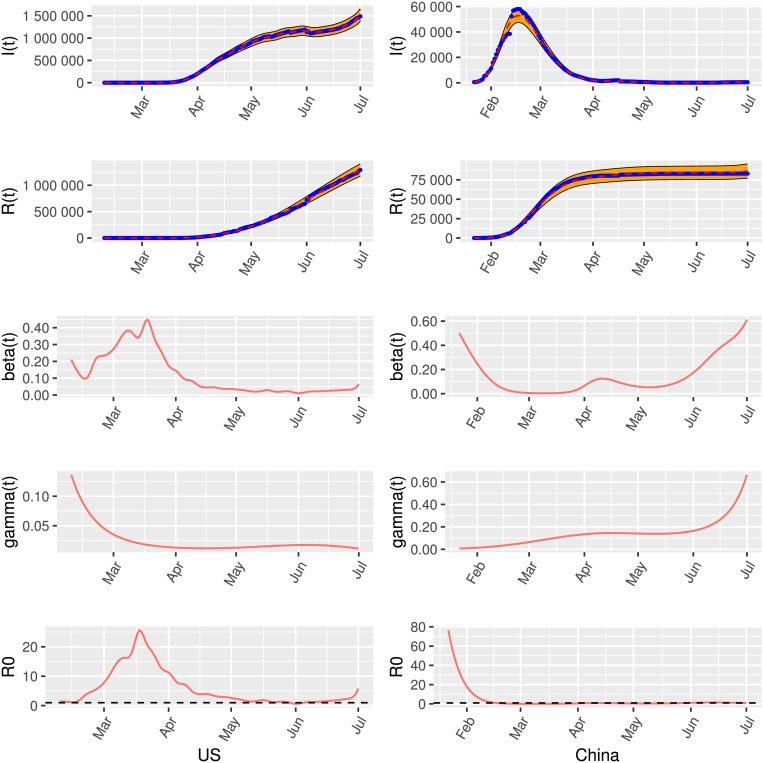
Estimated reproduction number $R_{0}\left(t\right)$ for selected countries based on the data up to June 30, 2020. The US (left) and China (right) are shown based on the data up to June 30, 2020. The blue dots and the red dashed curves represent the observed data and the model-based predictions, respectively, with 95% confidence interval.

**Fig 3:**
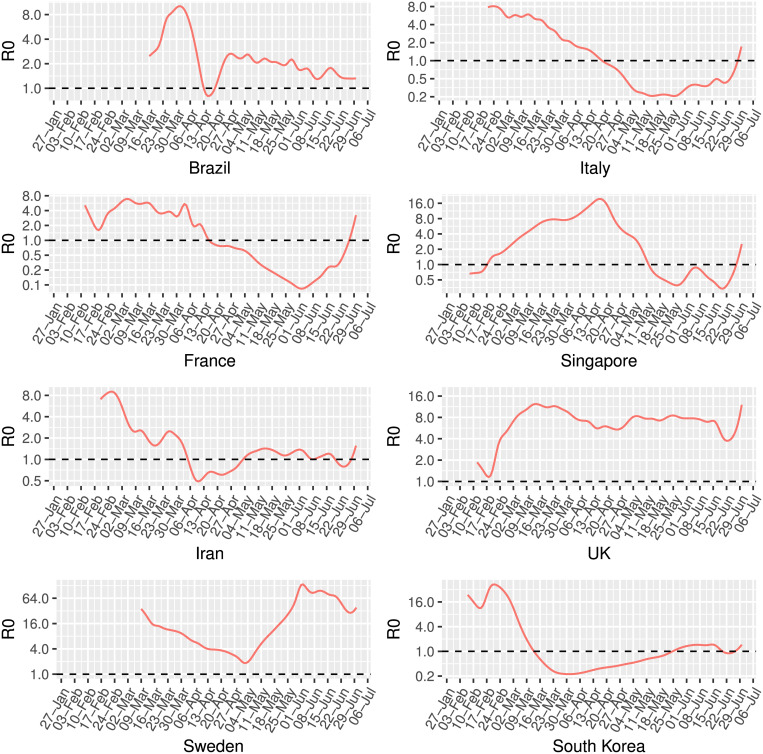
Estimated *I*(*t*), *R*(*t*), *β*(*t*), *γ*(*t*), and $R_{0}\left(t\right)$. The US (left) and China (right) are shown based on the data up to June 30, 2020. The blue dots and the red dashed curves represent the observed data and the model-based predictions, respectively, with 95% confidence interval.

Fig 2 shows sharp variations in transmission rates and removal rates across different time periods, indicating the time-varying nature of these rates. The estimated *I*(*t*) and *R*(*t*) overlapped well with the observed number of infectious and removed cases, indicating the reasonableness of the method. The pointwise 95% confidence intervals (in yellow) represent the uncertainty of the estimates, which may be due to error in reporting. Fig 3 presents the estimated time-varying reproduction number, $\hat{\beta }\left(t\right)\hat{\gamma }{\left(t\right)}^{-1}$, for several countries. The curves capture the evolving trends of the epidemic for each country.

In the US, though the first confirmed case was reported on January 20, 2020, lack of immediate actions in the early stage let the epidemic spread widely. As a result, the US had seen soaring infectious cases, and $R_{0}\left(t\right)$ reached its peak around mid-March. From mid-March to early April, the US tightened the virus control policy by suspending foreign travels and closing borders, and the federal government and most states issued mandatory or advisory stay-home orders, which seemed to have substantially contained the virus.

The high reproduction numbers with China, Italy, and Sweden at the onset of the pandemic imply that the spread of the infectious disease was not well controlled in its early phases. With the extremely stringent mitigation policies such as city lockdown and mandatory mask-wearing implemented in the end of January, China was reported to bring its epidemic under control with a quickly dropping $R_{0}\left(t\right)$ in February. This indicates that China might have contained the epidemic, with more people removed from infectious status than those who became infectious.

Sweden is among the few countries that imposed more relaxed measures to control coronavirus and advocated herd immunity. The Swedish approach has initiated much debate. While some criticized that this may endanger the general population in a reckless way, some felt this might terminate the pandemic more effectively in the absence of vaccines [50]. Fig 3 demonstrates that Sweden has a large reproduction number, which however keeps decreasing. The “big V” shape of the reproduction number around May 1 might be due to the reporting errors or lags. Our investigation found that the reported number of infectious cases in that period suddenly dropped and then quickly rose back, which was unusual.

Around February 18, a surge in South Korea was linked to a massive cluster of more than 5,000 cases [51]. The outbreak was clearly depicted in the time-varying $R_{0}\left(t\right)$ curve. Since then, South Korea appeared to have slowed its epidemic, likely due to expansive testing programs and extensive efforts to trace and isolate patients and their contacts [52].

More broadly, Fig 3 categorizes countries into two groups. One group features the countries which have contained coronavirus. Countries, such as China and South Korea, took aggressive actions after the outbreak and presented sharper downward slopes. Some European countries such as Italy and Spain and Mideastern countries such as Iran, which were hit later than the East Asian countries, share a similar pattern, though with much flatter slopes. On the other hand, the US, Brazil, and Sweden are still struggling to contain the virus, with the $R_{0}\left(t\right)$ curves hovering over 1. We also caution that, among the countries whose $R_{0}\left(t\right)$ dropped below 1, the curves of the reproduction numbers are beginning to uptick, possibly due to the resumed economy activities.

### 3.3 An interactive web application and R code

**Fig 4:**
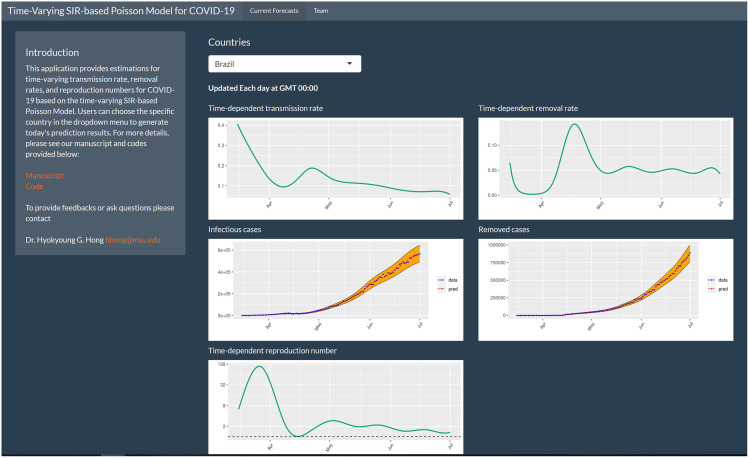
An illustration of the developed interactive web application.

We have developed a web application (https://younghhk.shinyapps.io/tvSIRforCOVID19/) to facilitate users’ application of the proposed method to compute the time-varying reproduction number, and estimated and predict the daily numbers of active cases and removed cases for the presented countries and other countries; see Fig 4 for an illustration.

Our code was written in R [53], using the bs function in the splines package for cubic B-spline approximation, the nlm function in the stats package for nonlinear minimization, and the jacobian function in the numDeriv package for computation of gradients and hessian matrices. Graphs were made by using the ggplot2 package. Our code can be found on the aforementioned shiny website.

## 4 Discussion

The rampaging pandemic of COVID-19 has called for developing proper computational and statistical tools to understand the trend of the spread of the disease and evaluate the efficacy of mitigation measures [54–57]. We propose a Poisson model with time-dependent transmission and removal rates. Our model accommodates possible random errors and estimates a time-dependent disease reproduction number, $R_{0}\left(t\right)$, which can serve as a metric for timely evaluating the effects of health policies.

There have been substantial issues, such as biases and lags, in reporting infectious cases, recovery, and deaths, especially at the early stage of the outbreak. As opposed to the deterministic SIR models that heavily rely on accurate reporting of initial infectious and removed cases, our model is more robust towards mis-specifications of such initial conditions. Applications of our method to study the epidemics in selected countries illustrate the results of the virus containment policies implemented in these countries, and may serve as the epidemiological benchmarks for the future preventive measures.

Several methodological questions need to be addressed. First, we analyzed each country separately, without considering the traffic flows among these countries. We will develop a joint model for the global epidemic, which accounts for the geographic locations of and the connectivity among the countries.

Second, incorporating timing of public health interventions such as the shelter-in-place order into the model might be interesting. However, we opted not to follow this approach as no such information exists for the majority countries. On the other hand, the impact of the interventions or the change point can be embedded into our nonparametric time-dependent estimates.

Third, the validity of the results of statistical models eventually hinges on the data transparency and accuracy. For example, the results of Chinazzi et al. [58] suggested that in China only one of four cases were detected and confirmed. Also, asymptomatic cases might have been undetected in many countries. All of these might have led to underestimation of the actual number of cases. Moreover, the collected data could be biased toward patients with severe infection and with insurance, as these patients were more likely to seek care or get tested. More in-depth research is warranted to address the issue selection bias.

Finally, our present work is within the SIR framework, where removed individuals include recovery and deaths, who hypothetically are unlikely to infect others. Although this makes the model simpler and widely adopted, the interpretation of the *γ* parameter is not straightforward. Our subsequent work is to develop a susceptible-infectious-recovered-deceased (SIRD) model, in which the number of deaths and the number of recovered are separately considered. We will report this elsewhere.

## 5 Conclusion

Containment of COVID-19 requires the concerted effort of health care workers, health policy makers as well as citizens. Measures, e.g. self-quarantine, social distancing, and shelter in place, have been executed at various phases by each country to prevent the community transmission. Timely and effective assessment of these actions constitutes a critical component of the effort. SIR models have been widely used to model this pandemic. However, constant transmission and removal rates may not capture the timely influences of these policies.

We propose a time-varying SIR Poisson model to assess the dynamic transmission patterns of COVID-19. With the virus containment measures taken at various time points, $R_{0}$ may vary substantially over time. Our model provides a systematic and daily updatable tool to evaluate the immediate outcomes of these actions. It is likely that the pandemic is ending and many countries are now shifting gear to reopen the economy, while preparing to battle the second wave of virus attack [59, 60]. Our tool may shed light on and aid the implementation of future containment strategies.

## Supporting information

S1 Appendix Details of optimization. (PDF)
