# Supplementary material for: Estimation of time-varying reproduction numbers underlying epidemiological processes: A new statistical tool for the COVID-19 pandemic
Source: PLoS One. 2020 Jul 21;15(7):e0236464. doi: 10.1371/journal.pone.0236464 (PMC7373269; doi:10.1371/journal.pone.0236464)
Supplement: S1 Appendix — (PDF) [file pone.0236464.s001.pdf]

Appendix: Details of Optimization

To minimize (10), we differentiate $\ell(\boldsymbol{\beta}, \boldsymbol{\gamma})$ with respect to $(\boldsymbol{\beta}, \boldsymbol{\gamma})$. Then $(\hat{\boldsymbol{\beta}}, \hat{\boldsymbol{\gamma}})$ solves the following estimating equations:

$$\begin{aligned}
\sum_{t=1}^{T} \left[\left\{ \frac{z_R(t)}{r(t)} - 1 \right\} \frac{\partial r(t)}{\partial \boldsymbol{\beta}} + \left\{ \frac{z_I(t)}{i(t)} - 1 \right\} \frac{\partial i(t)}{\partial \boldsymbol{\beta}} \right] &= 0, \\
\sum_{t=1}^{T} \left[\left\{ \frac{z_R(t)}{r(t)} - 1 \right\} \frac{\partial r(t)}{\partial \boldsymbol{\gamma}} + \left\{ \frac{z_I(t)}{i(t)} - 1 \right\} \frac{\partial i(t)}{\partial \boldsymbol{\gamma}} \right] &= 0,
\end{aligned}$$

where the involved partial derivatives can be computed recursively. Specifically, taking partial derivatives on the both sides of (7) yields that, for $t = 0, \ldots, T-1$,

$$\begin{aligned}
\frac{\partial i(t+1)}{\partial \boldsymbol{\beta}} &= [\{\beta(t)+1\}\{1-\gamma(t)\} - 2\beta(t)i(t)] \frac{\partial i(t)}{\partial \boldsymbol{\beta}} + i(t)\{1-\gamma(t)-i(t)\} \frac{\partial \beta(t)}{\partial \boldsymbol{\beta}}, \\
\frac{\partial i(t+1)}{\partial \boldsymbol{\gamma}} &= [\{\beta(t)+1\}\{1-\gamma(t)\} - 2\beta(t)i(t)] \frac{\partial i(t)}{\partial \boldsymbol{\gamma}} - i(t)\{\beta(t)+1\} \frac{\partial \gamma(t)}{\partial \boldsymbol{\gamma}}, \\
\frac{\partial r(t+1)}{\partial \boldsymbol{\beta}} &= \frac{\partial r(t)}{\partial \boldsymbol{\beta}} + \gamma(t) \frac{\partial i(t)}{\partial \boldsymbol{\beta}}, \\
\frac{\partial r(t+1)}{\partial \boldsymbol{\gamma}} &= \frac{\partial r(t)}{\partial \boldsymbol{\gamma}} + \gamma(t) \frac{\partial i(t)}{\partial \boldsymbol{\gamma}} + i(t) \frac{\partial \gamma(t)}{\partial \boldsymbol{\gamma}}.
\end{aligned}$$

Here, $\frac{\partial}{\partial \boldsymbol{\beta}} \beta(t) = \beta(t) \times \mathbf{B}(t)$, and $\frac{\partial}{\partial \boldsymbol{\gamma}} \gamma(t) = \gamma(t) \times \mathbf{B}(t)$, and $\frac{\partial}{\partial \boldsymbol{\beta}} i(0) = \frac{\partial}{\partial \boldsymbol{\gamma}} i(0) = \frac{\partial}{\partial \boldsymbol{\beta}} r(0) = \frac{\partial}{\partial \boldsymbol{\gamma}} r(0) = 0$ by using the initial conditions.
